# Focus on the Role of Inflammation as a Bridge between Ferroptosis and Atrial Fibrillation: A Narrative Review and Novel Perspective

**DOI:** 10.31083/j.rcm2504110

**Published:** 2024-03-25

**Authors:** Chenyang Jin, Zikan Zhong, Longzhe Gao, Xiaoyu Wu, Changzuan Zhou, Genqing Zhou, Shaowen Liu

**Affiliations:** ^1^Department of Cardiology, Shanghai General Hospital, Shanghai Jiao Tong University School of Medicine, 201620 Shanghai, China

**Keywords:** atrial fibrillation, ferroptosis, inflammation

## Abstract

In this comprehensive review, we examine the intricate interplay between inflammation, ferroptosis, and atrial fibrillation (AF), highlighting their significant roles in AF pathophysiology and pathogenesis. Augmented inflammatory responses are pivotal to AF, potentially leading to atrial remodeling and reentry phenomena by impacting calcium channels and atrial tissue fibrosis. A strong correlation exists between inflammatory cytokines and AF, underscoring the importance of inflammatory signaling pathways, such as NOD-like receptor thermal protien domain associated protein 3 (NLRP3) inflammasome, Nuclear Factor kappa B (NF-κB) signaling, and Tumor necrosis factor-α (TNF-α) signaling in AF development. Ferroptosis, a non-apoptotic regulated mode of cell death, has been widely studied in relation to cardiovascular diseases including heart failure, myocardial infarction, cardiomyopathy, and reperfusion injury. The interaction between ferroptosis and inflammation is complex and mutually influential. While significant progress has been made in understanding the inflammation-AF relationship, the role of inflammation as a conduit linking ferroptosis and AF remains underexplored. The specific pathogenesis and key molecules of atrial fibrosis caused by ferroptosis are still not fully understood. Here we review the role of inflammatory signaling in ferroptosis and AF. We elucidated the association between ferroptosis and AF, aiming to unveil mechanisms for targeted inhibition of atrial cell fibrosis and to propose novel therapeutic strategies for AF. This exploration is vital for advancing our knowledge and developing more effective interventions for AF, a condition deeply intertwined with inflammatory processes and ferroptotic pathways.

## 1. Introduction

Atrial fibrillation (AF) is a common atrial tachyarrhythmia affecting tens of thousands globally. This atrial arrhythmia characterized by mural thrombosis and impaired cardiac function, diminishing quality of life and potentially leading to major adverse cardiovascular events (MACE) [1]. Over the past 30 years (1990–2019), the global incidence of atrial fibrillation has increased dramatically, from 2,313,549 (95% UI 1,764,441–2,950,592) in 1990 to 4,720,324 (95% UI 3,644,331–5,961,597) in 2019 [1]. Stroke, one of the most serious complications of AF, negatively affects the quality of life of AF patients, resulting in a significant burden to patients and their families. One cohort study has confirmed an overall incidence of ischemic stroke in AF patients of 30.8 per 1000 person-years during follow-up [2]. Furthermore, a clinical trial found that earlier utility of direct oral anticoagulants could reduce stroke incidence by an estimated 2.8% per month [3]. With the application of antiarrhythmic drugs, radiofrequency catheter ablation and anticoagulants, AF and its complications can be effectively controlled [2, 3]. However, the underlying mechanism for the occurrence and maintenance of AF remains unclear.

Older age, obesity, inflammation, abnormal hormone secretion, and genetic alterations are all linked to AF development [4]. At present, a growing body of evidence suggests a significant association between inflammation and AF [5]. It has been reported that ‘NOD-like receptor thermal protien domain associated protein 3’ (NLRP3) inflammasome increases AF susceptibility in obese patients [6]. NLRP3 inflammasomes in cardiomyocytes may contribute to the onset and maintenance of AF by promoting ectopic activity or reentry [4]. As a biomarker representing systemic inflammation, levels of C-reactive protein (CRP) are considered to be a prognostic factor of AF and have been positively correlated with the occurrence of AF [7]. A cohort study showed that multiple systemic inflammatory markers, including CRP, neutrophils, and macrophages, were significantly and linearly associated with AF after adjusting for statistical confounding variables [8]. Furthermore, ferroptosis, an iron-dependent form of cell death, also plays an important role in inflammatory signaling pathways [9]. Some antioxidants have shown anti-inflammatory effects as ferroptosis inhibitors in animal models [10]. Research also suggests that ferroptosis may affect tissue fibrosis through inflammation or the immune response [11]. In experimental studies, rats with chronic alcohol intake showed increased AF vulnerability; inhibiting ferroptosis reduced, suggesting a role for ferroptosis in the initiation of AF via atrial myocarditis [12]. These findings prompted our investigation into the role of ferroptosis and inflammation in the molecular mechanism for the development of AF. We sought to find a new molecular basis for the occurrence of AF, to provide a safer and more effective treatment for AF patients.

## 2. Overview of Ferroptosis

Ferroptosis is a type of cell death distinct from autophagy, apoptosis, and necroptosis, and its definition was first proposed by Brent R. Stockwell in 2012 [13]. Ferroptosis is an iron-dependent, non-apoptosis-regulated, oxidative cell death [13]. The main characteristics of ferroptosis include iron accumulation, and alterations in mitochondrial morphology, amino acid metabolism, lipid peroxidation (LPO) and other biochemical changes [13]. There are three critical events involved with ferroptosis: iron accumulation, glutathione (GSH) depletion, and LPO. These biochemical changes are the root cause of ferroptosis and other diseases. The immunological characteristics of ferroptosis are damage-associated molecular patterns (DAMPs) that release pro-inflammatory mediators such as high-mobility group protein B1 (HMGB1) [9, 13, 14]. This is one of the mechanisms by which ferroptosis causes tissues and cells to increase the inflammatory response. We summarize the characteristics and differences between ferroptosis and other types of cell death in Table 1.

**Table 1.** **Characteristics of different forms of cell death**.

Cell death type	Characteristics of microstructure changes	Biochemical characteristics	Key regulatory factors	Immune features
Ferroptosis	normal nuclear; mitochondrial shrinkage; darker mitochondria; rupture of the outer mitochondrial membrane; decreasing or vanishing of mitochondrial cristae	iron overload; LPO; abnormal amino acid metabolism; reduced glutathione	glutathione peroxidase 4 (GPX4); SLC7A11; p53; acyl-CoA synthetase long-chain family member 4 (ACSL4); erastin	pro-inflammatory
Apoptosis	formation of apoptotic bodies; nuclear fragmentation; condensation of chromatin; plasma membrane blebbing	activation of caspases; fragmentation of oligonucleotide DNA; bare phosphatidylserine; reduced mitochondrial membrane potential	BCL2 family; caspase; BAX; BAK; p53	anti-inflammatory
Autophagy	formation of autophagosomes; aggregation of double-membraned autophagic vesicles	conversion of LC3-I to LC3-II	LC3; ATG5/7; mammalian target of rapamycin (mTOR)	anti-inflammatory
Necroptosis	swelling of cells; condensation of chromatin; rupture of the plasma membrane	decreased ATP levels; accumulation of reactive oxygen species (ROS); phosphorylation of RIPK1/3 and MLKL; release of DAMPs	RIPK1; RIPK3; MLKL	pro-inflammatory
Pyroptosis	rupture of the plasma membrane; swelling of organelles; mitochondria whose integrity was not affected	activation of caspase-1; release of proinflammatory cytokines	caspase-1; gasdermins; NLRP3; GPX4	pro-inflammatory

LPO, lipid peroxidation; SLC7A11, solute carrier family 7 member 11; BCL2, B-cell lymphoma 2; BAX, BCL2 associated X protein; BAK, BCL2 antagonist/killer 1; LC3, microtubule associated protein light chain 3; ATG5/7, autophagy related gene 5/7; DAMPs, damage-associated molecular patterns; RIPK1, recombinant receptor interacting serine/threonine kinase 1; RIPK3, recombinant receptor interacting serine/threonine kinase 3; MLKL, mixed lineage kinase domain-like protein; NLRP3, NOD-like receptor thermal protien domain associated protein 3.

To date, ferroptosis has been shown to be involved in cell death in Alzheimer’s disease, cancer, ischemia reperfusion (including stroke and myocardial infarction), liver fibrosis, renal tubule fibrosis and other diseases [15, 16, 17, 18, 19, 20]. In view of the many characteristics of ferroptosis and its harmful effects, particularly, since the mechanism by which it caused fibrosis in cardiomyocytes is not well understood, a detailed review of the link between ferroptosis and the heart is warranted.

### 2.1 Cardiomyocyte Iron Homeostasis

**Fig. 1.**
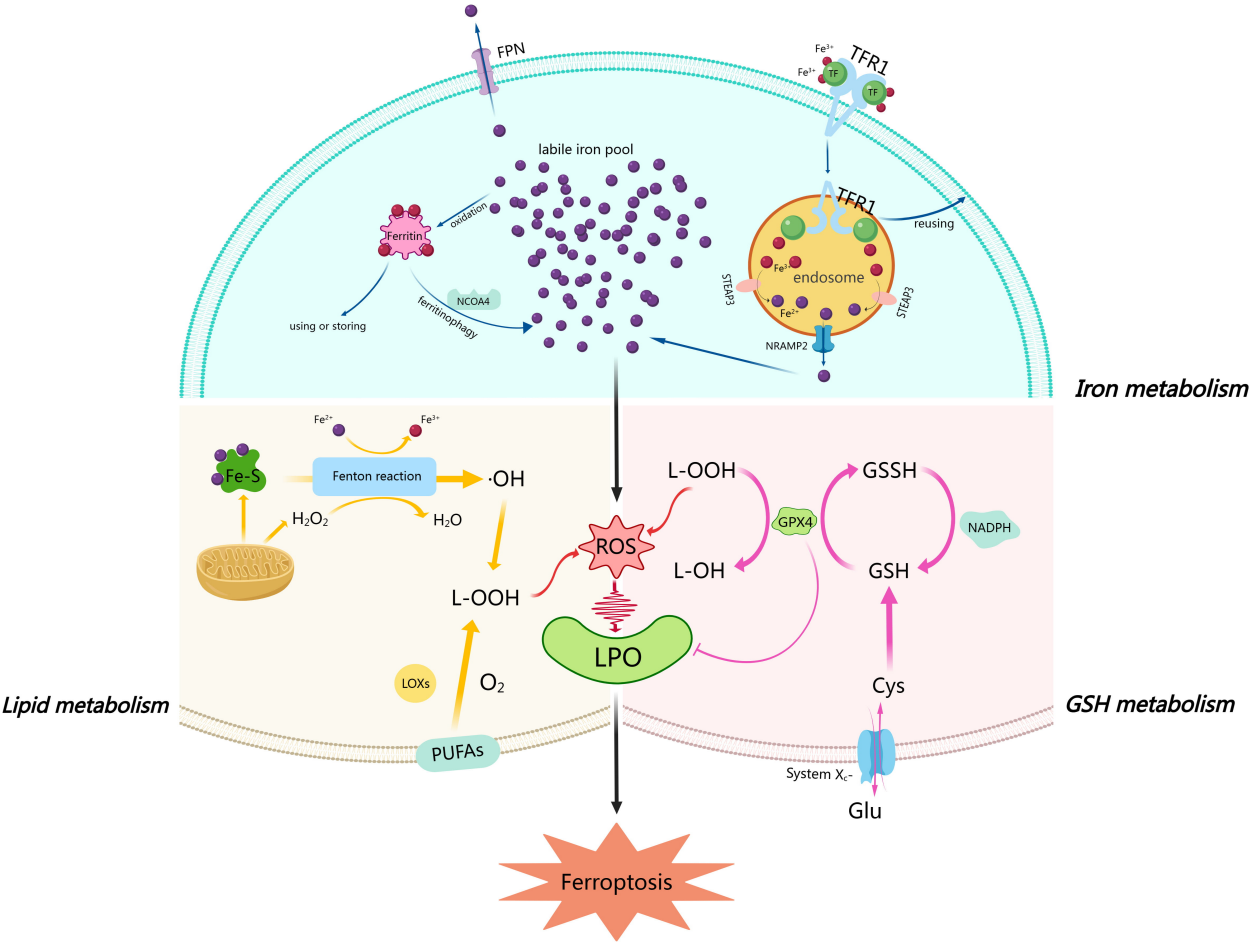
**Metabolic Pathways associated with ferroptosis in cardiomyocytes**. Iron metabolism and cell signaling in cardiomyocytes: Nonheme iron is transported into the cell by TF and its receptor, TFR1. Subsequently, the endosome is acidified by ATPases, inducing the STEAP metalloreductase family to reduce ferric to ferrous iron. Ferrous iron is released into the cytoplasm by NRAMP2, while TF and TFR1 are transported back to the cell membrane for reuse. Ferrous iron that is transported to the cytoplasm is oxidized to ferric iron, which is bound to ferritin and used in enzymatic reactions or stored for later use. Saturated ferritin is degraded by NCOA4-mediated autophagy, a process known as ferritinophagy, and eventually, the ferrous iron produced by degradation and the ferrous iron released from endosomes form an intracellular labile iron pool. In GSH metabolism, the $X_{\text{𝑐}}$^-^ system comprises of two subunits (SLC3A2 and SLC7A11) and functions as a cystine/glutamate antiporter on the cell membrane. It is responsible for transporting cystine into the cell and glutamate out of the cell. Cystine is broken down inside the cell to cysteine, which is used to synthesize GSH. GSH is converted to GSSH catalyzed by GPX4, and at the same time, L-OOH is converted to L-OH. In Lipid metabolism, PUFAs within the cell membrane are catalyzed by LOXs and oxidized to L-OOH. The mitochondria produce Fe-S and $H_{2}$$O_{2}$. In the Fenton reaction, ferrous iron is oxidized, and $H_{2}$$O_{2}$ is dehydrogenated to $H_{2}$O. The Fenton reaction generates hydroxyl radicals (⋅OH), a type of reactive free radical, which are eventually converted to L-OOH. The L-OOH produced in this process, along with that generated by glutathione peroxidase 4 (GPX4) metabolism, further exacerbates ROS and LPO accumulation within the cell. TF, transferrin; TFR1, transferrin receptor 1; STEAP3, six-transmembrane epithelial antigen of prostate 3; NRAMP2, natural resistance associated macrophage protein 2; NCOA4, nuclear receptor coactivator 4; GSH, glutathione; SLC3A2, solute carrier family 3 member 2; SLC7A11, solute carrier family 7 member 11; GSSH, glutathione disulfide; GPX4, glutathione peroxidase 4; L-OOH, lipid hydroperoxide; L-OH, lipid alcohol (or lipid hydroxide); PUFAs, polyunsaturated fatty acids; LOXs, lipoxygenases; Fe-S, iron-sulfur; $H_{2}$$O_{2}$, hydrogen peroxide; $H_{2}$O, water; ⋅OH, hydroxyl radical; ROS, reactive oxygen species; LPO, lipid peroxidation; FPN, ferroportin.

In general, the regulation of iron homeostasis in cardiomyocytes is similar to that in other systemic cells of the body (Fig. 1).

Physiologically, transferrin (TF) binds two molecules of ferric ions, and subsequently, they bind to the receptor of TF, transferrin receptor protein 1 (TFR1) [21]. It is this process that initiates and mediates the uptake of ferric irons by cells. In addition to this pathway, iron influx into cells can occur via the DMT-1 protein, L/T-type calcium channels at the cardiac plasma membrane, and zinc transporters [21]. Subsequently, ferric irons undergo a reduction reaction mediated by the metal reductase STEAP3 (six-transmembrane epithelial antigen of prostate) to become ferrous irons [22]. Ferrous irons are detached from transferrin in endocytic lysozymes and released into the cytoplasm by natural resistance associated macrophage protein 2 (NRAMP2), involved in the composition of labile iron pools in the cytosol [21]. Apo-transferrin and TFR1 are recycled and transported back to the cell surface.

The ferrous iron in the cytoplasm binds to the ferritin heavy chain (FTH), oxidizes to a ferric state, and forms ferritin-bound iron [22]. Iron, on the other hand, is released from the FTH by nuclear receptor coactivator 4 (NCOA4) mediated degradation of ferritin, a process known as ferritinophagy [22]. Through the above cycle, cells maintain iron homeostasis by storing excess iron ions or releasing iron ions into the cytoplasm for use when iron is needed. In addition, the excess iron can also be removed from the cell through ferroportin (FPN) [22]. In physiological conditions, labile iron is kept at very low levels, preventing the production of excessive reactive oxygen species (ROS) [21]. Nevertheless, intracellular iron overload can significantly increase the labile iron pool, resulting in the hazardous accumulation of ROS and ferroptosis.

### 2.2 Amino Acid Metabolism in Ferroptosis

The amino acid metabolism pathway in ferroptosis prominently features GSH, a tripeptide composed of cysteine, glutamate, and glycine, and is ubiquitous in cells. GSH plays a crucial role in reducing harmful per-oxidants generated by cell metabolism to harmless lipid alcohols [23]. It exists in two forms: a reduced form (GSH) and an oxidized form (G-S-S-G, glutathione disulfide) [23]. Extracellular cystine and intracellular glutamate enter and exit the cell in a 1:1 ratio via the cystine/glutamate reverse transporter respectively, named system $X_{\text{𝑐}}$^-^, containing two subunits (solute carrier family 3 member 2 (SLC3A2) and solute carrier family 7 member 11 (SLC7A11)) [24]. The cystine transported into the cell is used for the biosynthesis of GSH [23]. As a vital functional cofactor of glutathione peroxidase 4 (GPX4), GSH is converted to GSSH catalyzed by GPX4, a process synchronized to the conversion of L-OOH to L-OH [23, 25]. Concurrently, NADPH is converted to ${\mathrm{NADP}}^{+}$, which is catalyzed by glutathione reductase [23]. Notably, GPX4 can maintain the balance of intracellular redox state and can also remove excessive accumulation of harmful peroxidation products in cells [24, 25]. Thus, GPX4 plays an integral role in preventing ferroptosis in cells [24, 25]. Depletion of GSH or reduced glutathione synthesis (e.g., cysteine deficiency) leads to reduced or inactivated GPX4 content, which in turn promotes ferroptosis in cells [22, 24, 25, 26]. In addition, Erastin can deplete intracellular cystine by inhibiting system $X_{\text{𝑐}}$^-^, eventually depleting GSH and inactivating GPX4, leading to ferroptosis [13]. In conclusion, GPX4 is an important antioxidant in cells and plays a unique role in the prevention and treatment of ferroptosis. Additionally, some metabolic pathways, such as mevalonate, are also involved in the process of ferroptosis through the production of squalene, isopentenyl pyrophosphate, coenzyme Q10 and other biomolecules with anti-pyrophosphate activity, which also affect ferroptosis [14]. Amino acid metabolism and lipid metabolism in ferroptosis is shown Fig. 1.

### 2.3 LPO in ferroptosis

Phospholipids, an important component in maintaining the fluidity of the cell membrane, is also damaged through ferroptosis [25, 26]. Polyunsaturated fatty acids (PUFAs) in phospholipids, such as docosahexaenoic acid and arachidonic acid (AA), are the main substances that mediate intracellular signal transduction and are also vulnerable to peroxidation damage during ferroptosis [25]. It has been shown that the acyl-CoA synthetase long-chain family member 4 (ACSL4) and lysophosphatidylcholine acyltransferase 3 (LPCAT3) are involved in the biosynthesis of polyunsaturated fatty acids in phospholipids [27]. Phosphatidylethanolamines combine with AA and adrenal acid (AdA) to synthesize PUFAs under the catalysis of ACSL4 and CoA; and free PUAFs become bound to membrane phospholipids through the activity of LPCAT3 [28, 29]. Acylation of PUFAs by ACSL4, which increases the PUFA content of phospholipids, is thought to be a specific factor for ferroptosis [27]. After synthesis, PUAFs become embedded into the phospholipids of the cell membrane, and are subsequently oxidized by lipoxygenases (LOXs) to become L-OOH [25]. Compounds including Fe-S and $H_{2}$$O_{2}$ supplied by mitochondria participate in the Fenton reaction in the cell, in which ${\mathrm{Fe}}^{2+}$ is oxidized to ${\mathrm{Fe}}^{3+}$ and the reactive free radical (⋅OH) is generated, which in turn is converted to L-OOH [30]. Normally, the intracellular production of trace lipid hydrogen can be reduced to lipid alcohols by GPX4, thereby maintaining the stability of cells to avoid the damage of ROS. However, ferroptosis is driven by iron-dependent LPO, and plays a pivotal role linking ferroptosis to various diseases and abnormal metabolic states [31]. When lipid metabolism in cardiomyocytes is altered through the lipoxygenase and Fenton reaction pathways, malignant LPO will occur in the cells, and GPX4 will become depleted, resulting in ferroptosis [28, 30, 31]. Moreover, LPO could damage the cardiac cell membrane by contributing to ferroptosis [26, 28]. LPO is also involved in the inflammatory response, which may play an important molecular and pathological role in the occurrence of AF, and the details will be explained in the next section.

## 3. The Role of Ferroptosis and Inflammation in AF

The relationship between ferroptosis and fibrosis is complex. At present, it has been shown that ferroptosis can lead to tissue fibrosis in a variety of organs. A study found that the alveolar type II cells of bleomycin induced mice had iron and collagen deposition, suggesting that ferroptosis promoted the progression of pulmonary fibrosis [32]. Another study demonstrated that for simple hepatic steatosis, ferroptosis was a leading factor that led to non-alcoholic steatohepatitis, which included liver injury, infiltration of immune cells, and inflammatory cell aggregation [33], resulting in liver inflammation and fibrosis. It has also been reported that administering ferristatin-1 to mice with acetaminophen could reverse liver fibrosis [34]. However, it seems that ferroptosis is a double-edged sword for liver fibrosis. It was found that curcumol inhibited liver fibrosis by inducing ferroptosis in hepatic stellate cells [35]. It was generally believed that in parenchymal cells, ferroptosis may exacerbate tissue fibrosis; but in the case of muscle fiber cells, ferroptosis may inhibit the progression of fibrosis [29]. Therefore, there is an important link between ferroptosis and fibrosis in the process of disease formation in these organs.

A recent study revealed a notable association between cardiomyocyte fibrosis and ferroptosis in patients with AF, with findings indicating a higher incidence of fibrosis in this group compared to controls [36]. The specific mechanisms linking atrial myocyte fibrosis and AF to ferroptosis, as well as the role of inflammation in this process, remain underexplored. A study highlighted that markers of inflammation, oxidative stress, and fibrosis were significantly associated in AF patients [37]. Specifically, myeloperoxidase (odds ratio [OR] = 1.012, *p* = 0.014) and high-sensitivity CRP (OR = 1.265, *p* = 0.026) were independently correlated with AF compared to controls [37]. Another experimental study also found that the administration of colchicine to experimental rats inhibited Interleukin 1β (IL-1β)-induced IL-6 release and prevented subsequent atrial fibrosis [38]. In the next section, we present a detailed review of ferroptosis, the inflammatory response and AF.

### 3.1 Ferroptosis and Inflammation

**Fig. 2.**
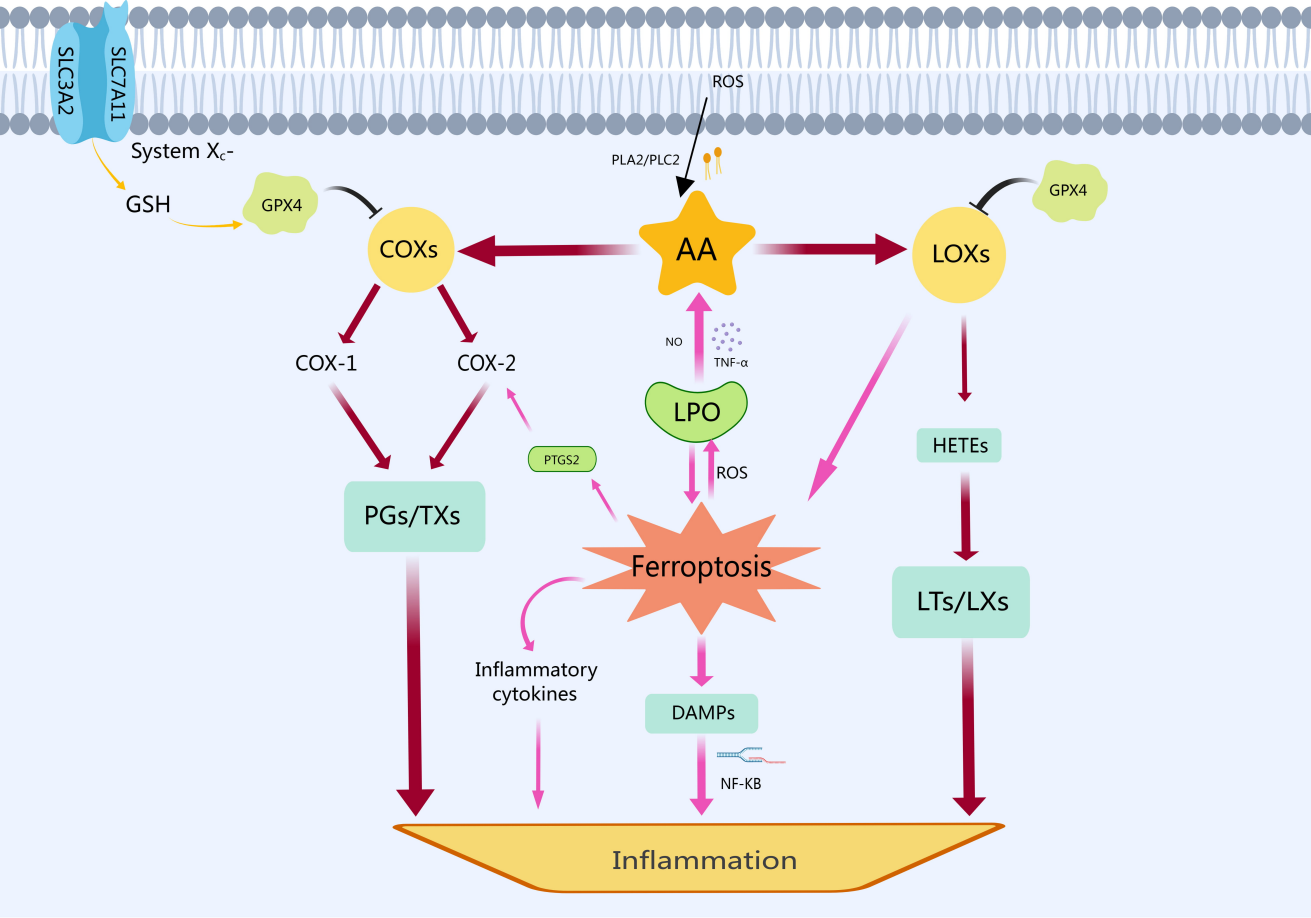
**The relationship between ferroptosis and inflammation**. In response to cellular stress or stimulation, phospholipase A2 (PLA2) and phospholipase C2 (PLC2) break down cell membrane phospholipids into AA. The polyunsaturated fatty acid AA is produced by catabolism under the stimulation of cellular oxidative stress and LPO. Subsequently, cyclooxygenases (COXs) metabolize AA to prostaglandins (PGs) and thromboxanes (TXs), which can induce an inflammatory response. Simultaneously, LOXs convert AA into hydroxyeicosatetraenoic acids (HETEs), leading to the production of leukotrienes (LTs) and lipoxins (LXs). Additionally, LOXs can also exacerbate ferroptosis by enhancing LPO and releasing ROS. As the core of ferroptosis, LPO perpetuates a vicious cycle by promoting further AA decomposition through substances like nitric oxide (NO) and tumor necrosis factor-alpha (TNF-α), exacerbating ferroptosis. In addition, ferroptosis can also promote the metabolism of COX-2-induced AA by upregulating prostaglandin-endoperoxide synthase 2 (PTGS2), the gene encoding COX-2. Ferroptotic cells release DAMPs and inflammatory cytokines, activating nuclear factor kappa-light-chain-enhancer of activated B cells (NF-κB) to promote aseptic inflammation. Consequently, PGs, TXs, LTs, LXs, DAMPs and other inflammatory cytokines contribute to inflammation. ROS, reactive oxygen species; AA, arachidonic acid; PLA2, phospholipase A2; PLC2, phospholipase C2; COX, cyclooxygenase; PTGS2, prostaglandin-endoperoxide synthase 2; PG, prostaglandin; TXs, thromboxane synthetase; HETE, hydroxyeicosatetraenoic acid; LT, leukotriene; LX, lipoxin; SLC3A2, solute carrier family 3 member 2; SLC7A11, solute carrier family 7 member 11; GSH, glutathione; GPX4, glutathione peroxidase 4; NF-κB, nuclear factor kappa B; TNF-α, tumor necrosis factor-α; LPO, lipid peroxidation; LOXs, lipoxygenases.

An additional characteristic of ferroptosis is a large release of oxidized lipid mediators [39]. Undergoing ferroptosis makes cells more immunogenic, as they release DAMPs and pro-inflammatory factors that drive the tissue environment toward an inflammatory state [9, 40]. As a DAMP, HMGB1 plays a vital role in the formation of and pathological mechanism behind inflammation, as well as amplifying the inflammatory response [14]. Once released by the cell, HMBG1 gains immune-stimulating properties and acts as an adjuvant, promoting an immune response by binding to pattern recognition receptors and activating inflammation [9, 14, 40]. Furthermore, neutralizing anti-HMBG1 antibodies attenuates the macrophage inflammatory response induced by ferroptosis [41]. A possible association between ferroptosis and inflammation is shown in Fig. 2.

Previously, we discussed the metabolic pathways of ferroptosis, highlighting LPO as the underlying mechanism driving inflammation associated with ferroptosis. It is well known that PUFAs are the main components of cell membranes. PUFAs may play a crucial role in regulating inflammatory signaling and antioxidant pathways [42]. Increased intake of eicosapentaenoic acid and docosahexaenoic acid is beneficial in reducing the incidence of diseases characterized by elevated inflammation (including cardiovascular disease) [42]. PUFAs and their related metabolic enzymes have become key cellular and molecular factors resulting in inflammation [9]. PUFAs are also the most sensitive lipid in the process of ferroptosis [43, 44]. In one study, phosphatidylethanolamine (PE) was identified as a lipid associated with AF [45]. The authors suggested that PE impaired mitochondria and aggravated LPO by promoting the increase of oxidative products, triggering ferroptosis in atrial cells, and participating in atrial fibrosis [45]. Atrial cells with iron overload are in a state of cellular stress, accompanied by increased intracellular ROS, while AA is released by phospholipase C (PLC) or phospholipase A2 (PLA2) from phospholipids of the cell membrane. Additionally, AA can be oxidized by LOX, cyclooxygenase (COX) and cytochrome P450 monooxygenase, and metabolized into a variety of bioactive inflammatory mediators, such as leukotrienes (LTs), prostaglandins (PGs), and hydroxyeicosatetraenoic acid [9, 14]. These substances all contribute to the inflammatory response.

There are six LOX isoforms in the human body [43], which play an essential role in the cellular oxidation of ferroptosis. This family of lipid-peroxidases that catalyzes the peroxidation of PUFAs to produce lipid hydrogen peroxide products [43]. Furthermore LOX oxidizes AA into hydroperoxyl-intermediates including hydroperoxyeicosatetraenoic acids (HPETE) and hydroxyeicosatetraenoic acids (HETE), which are then metabolized into leukotrienes (LTs), lipotoxins (LXs) and other pro-inflammatory substances [9, 14, 44]. LOX, in addition to acting through enzyme-catalyzed lipid peroxidation, also alerts immune cells through LOX-derived proinflammatory metabolites, including LTB4/LTC4/LTD4 and LTE4, indirectly sensitizing the cell to ferroptosis [46].

COX is a key rate-limiting enzyme for the conversion of AA to PG. Two isoenzymes COX-1 and COX-2 have been found to play a key role in this process [47]. While COX-1 is widely expressed in various cell types, COX-2 is typically less expressed in normal tissues and mainly functions in activating macrophages and other inflammatory cells [47]. The gene *prostaglandin endoperoxide synthase 2* (*PTGS2*) encodes COX enzymes, which are responsible for metabolizing AA found within cell membranes into a range of inflammatory factors including prostaglandin E2 (PGE2) [9]. Studies have shown that ferroptosis can directly increase the expression of PTGS2, and then encode more COX-2, accelerating AA metabolism, and ultimately promoting the secretion of inflammatory factors [9, 48]. Thus, inflammation induced by ferroptosis may be related to the increase of PTGS2 expression and PGE2 release.

Inflammation is also related to oxidative stress, which can trigger a range of pro-inflammatory (such as tumor necrosis factor-α [TNF-α]) and transcription factors (such as NF-κB) [14]. Due to the deficiency or inactivation of GPX4, or the introduction of an inducer of ferroptosis, there are increased levels of lipid-ROS and LPO in the cell, resulting in ferroptosis [49]. It is known that GPX4 can play a role in cytoprotection by reducing the production of cellular lipid hydroperoxide [50]. Furthermore, GPX4 alleviates ferroptosis and inflammation by inhibiting the oxidation of AA and activation of the NF-κB pathway [51]. This also reduces the production of inflammatory factors caused by LPO, which is known to polarize macrophages by increasing modified low-density lipoproteins (LDL), promoting inflammation [14]. Ferroptosis can also lead to the polarization of macrophages into the M1 type through increases of ROS, TNF-α and IL-1β [52]. Notably, the M1 macrophages have pro-inflammatory properties [52]. As previously discussed, the decrease in intracellular GPX4 content and the imbalance of intracellular redox state aggravate the sensitivity of cells to ferroptosis and simultaneously increase the production of intracellular inflammatory factors. Excess ROS consumes the pool of intracellular antioxidants, consequently increasing inflammation, LPO production, and ferroptosis aggravation producing a cytotoxic feedback loop [49, 51, 52]. By inhibiting LPO that is central to ferroptosis (such as inhibiting PE), the ferroptosis inflammatory response can be suppressed, and decreasing AF progression [45]. Consequently, these enzymatic targets may lead to a potential mechanism for the prevention and treatment of AF [45].

### 3.2 Ferroptosis, Inflammation and AF

**Fig. 3.**
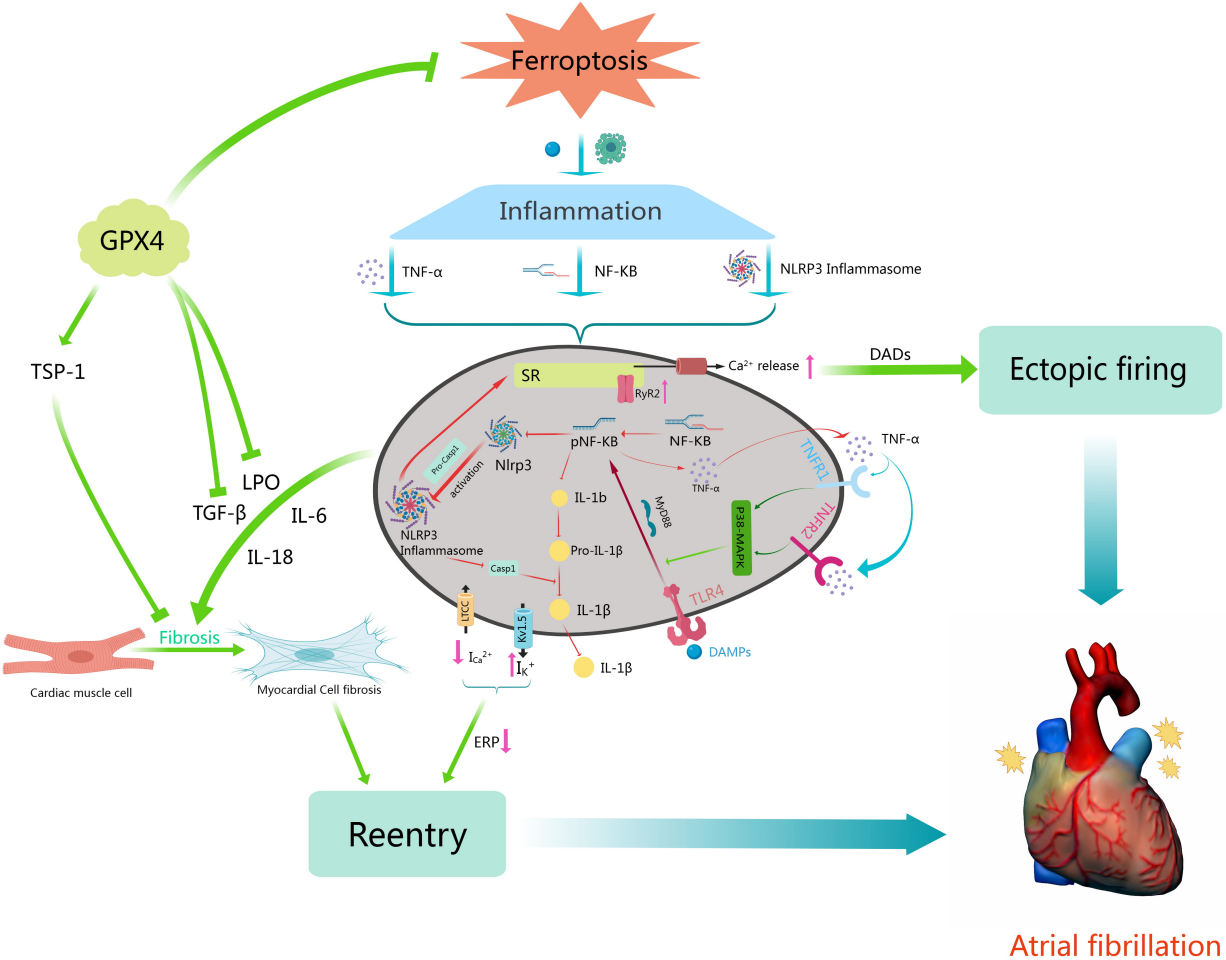
**The interplay between inflammation, ferroptosis, and AF. **Ferroptosis can influence atrial cells through inflammation, with atrial inflammation being a crucial contributor to both the onset and persistence of AF. This figure outlines a possible mechanism by which intracellular ferroptosis induces and sustains AF through inflammatory pathways. Key factors in AF induction include reentry and ectopic firing, which are associated with the abnormal release of ${\mathrm{Ca}}^{2+}$, excessive intake of $K^{+}$, and the release of various inflammatory factors resulting in atrial fibrosis. Central to this process are TNF-α, NF-κB, and NLRP3 inflammasome, each playing a vital and irreplaceable role. TSP-1, thrombospondin 1; SR, sarcoplasmic reticulum; GPX4, glutathione peroxidase 4; IL-6, interleukin 6; IL-18, interleukin 18; TGF-β, transforming growth factor-β; LPO, lipid peroxidation; ERP, effective refractive period; IL-1β, interleukin 1β ; DAMPS, damage-associated molecular patterns; TLR4, toll-like receptor 4; Myd88, myeloid differentiation factor 88; P38-MAPK, p38 mitogen activated protein kinases; TNFR1/2, tumor necrosis factor-α receptor type 1/2; NF-κB, nuclear factor kappa B; TNF-α, tumor necrosis factor-α; NLRP3, NOD-like receptor thermal protien domain associated protein 3; RyR2, ryanodine receptor 2; DADs, depolarization after delays; Casp1, caspase-1; AF, atrial fibrillation.

It has been suggested that inflammatory atrial cardiomyopathy, also known as atrial myocarditis, increases AF susceptibility through tissue fibrosis, electrophysiological remodeling, autonomic nerve remodeling, and other mechanisms [53]. At present, a number of studies found that inhibition of NF-κB and COX-2 signaling may reduce the release of PGE2, NO, and other inflammatory mediators, which could exert anti-inflammatory activity [54, 55, 56]. One study also found that PGE2 regulated the polarization of macrophages through the NF-κB signaling pathway, thereby affecting the inflammatory response [57]. Furthermore, signaling pathways such as NF-κB, TNF-α and NLRP3 inflammasome are central to the inflammatory response orchestrated by innate immune cells. They are mainly responsible for the maturation and release of cytokines, and can also independently participate in the pathogenesis of AF [53, 56, 57, 58]. By targeting the NF-κB signaling pathway, ferroptosis related diseases could potentially be reversed [58, 59]. In addition, LPO is implicate in activating the NLRP3 inflammasome [58], further emphasizing the interplay between inflammasomes, ferroptosis, and AF. As shown in Fig. 3, NF-κB, TNF-α, and NLRP3 inflammasomes exemplify key inflammatory pathways, highlighting their role in linking ferroptosis and AF. Table 2 (Ref. [36, 45, 60, 61, 62, 63]) summarizing the relevant literature currently reported on ferroptosis and AF.

**Table 2.** **Summary of available laboratory evidence on ferroptosis and AF**.

	The recruited patient group	Type of experimental tissue	Additional intervention factor	Key findings and results	References
1	Patients with valvular disease who underwent heart valve surgery.	human tissues	——	1. Left atrial tissue showed significant fibrosis in the AF group compared to the sinus rhythm group.	[36]
				2. There was a significant increase in iron particles in the left atrial appendage tissue stained with Prussian blue in AF group.	
				(Control group 13.35 ± 1.81% vs AF group 25.8 ± 2.72%, n = 8, *p* = 0.0018)	
				3. Ferroptosis was associated with atrial fibrosis in group of AF.	
				(r = 0.7763, *p* = 0.0004)	
2	Patients with AF undergoing elective heart valve replacement and mice treated with PE.	human tissues and animal tissues	PE treatment group	1. PE was a characteristic differential lipid in the AF group.	[45]
				2. PE promoted cardiomyocyte death by increasing mitochondrial damage and oxidative stress.	
				3. PE induced ferroptosis by inhibiting GPX4 and increasing ACSL4 expression.	
				4. Ferroptosis played an important role in atrial fibrosis induced by AngII alone and AngII combined with PE.	
3	Fecal microbiota from mice fed a high-fat diet was transplanted into mice fed a normal diet.	animal tissues	High-fat diet administration	1. High-fat diet changed the composition of gut microbiota, potentially increasing susceptibility to AF through systemic inflammation.	[60]
				2. Pathway enrichment analysis showed that ferroptosis in high-fat diet group was significantly associated with inflammatory pathways such as NF-κB signaling pathway (*p* = 0.00045).	
				3. The ferroptosis-related protein GPX4 was decreased and PTGS2 was increased in high-fat diet group.	
4	A rat model of endotoxemia established by intraperitoneal injection of LPS.	animal tissues	Sepsis	1. The induction rate and duration of AF in the LPS group were significantly higher than in the control group.	[61]
				2. The expression of GPX4 was decreased and the expression of PTGS2 was increased in LPS group.	
				3. In the LPS group, total iron was increased and Fpn protein was decreased.	
5	Excessive ethanol-treated mouse model.	animal tissues	Excessive ethanol administration	1. Induction and duration of AF were significantly increased in the excess ethanol treatment group.	[62]
				2. Atrial fibrosis was found in the excess ethanol group.	
				3. PTGS2, P53 and ACSL4 were significantly increased in the excess ethanol treatment group.	
				4. Iron accumulation was observed in the excess ethanol treated group by Prussian blue staining.	
6	A beagle model of AF, induced by placing a pacemaker in the carotid artery.	animal tissues	——	1. The transcription and translation levels of GPX4 and SLC7A11 were significantly decreased in the pacing group.	[63]
				2. An increase in total iron in atrial tissue was observed in the rapid pacing group.	
				3. There was a significant accumulation of malondialdehyde in atrial tissue in the pacing group.	

AF, atrial fibrillation; PE, phosphatidylethanolamine; GPX4, glutathione peroxidase 4; ACSL4, acyl-CoA synthetase long-chain family member 4; NF-κB, nuclear factor kappa B; PTGS2, prostaglandin-endoperoxide synthase 2; LPS, lipopolysaccharide; SLC7A11, solute carrier family 7 member 11.

#### 3.2.1 NF-κB Pathway

The classical transcription factor NF-κB plays a vital role in both innate immunity and the inflammatory response [59, 64]. There are two distinct activation pathways of the NF-κB signaling pathway, canonical and non-canonical NF-κB pathways [59]. The canonical NF-κB pathway is rapidly activated in innate immune cells by a variety of signals such as innate pattern recognition receptors (PRRs) and pro-inflammatory cytokines receptors [59]. The PRRs are usually expressed on innate immune cells (such as macrophages, neutrophils, and monocytes). The PRRs recognize DAMPs released by damaged cells and consequently induce the expression of pro-inflammatory cytokines such as TNF-α and IL-1 [59]. Toll-like receptors are typical PRRs, and the Toll-like receptor 4 (TLR4) - myeloid differentiation factor 88 (Myd88) pathway is the most common activation mechanism of the NF-κB pathway [4]. Thus, activation of the canonical NF-κB pathway induced the innate immune system to produce pro-inflammatory cytokines such as TNF-α and IL-1β, which in turn further activated the canonical NF-κB pathway of other cells and amplified the inflammatory cascade reaction.

The NF-κB signaling pathway also plays an important role in the pathogenesis of AF. A recent study concluded that local inflammation, which occurs during atrial remodeling, was conducive to the production and maintenance of AF [65]. Furthermore, NF-κB was significantly associated with TNF-α and IL-6 [65]. It was also reported that transforming growth factor (TGF) plays a central role in the formation and maintenance of atrial fibrosis [66]. It has been previously suggested that in glioblastomas, NF-κB activated transforming growth factor-β (TGF-β)/Smad via miR-148a, promotes the proliferation of glioblastoma cells [67]. It was suggested that CRP itself can activate TLR4 and induce its interaction with TGF-β/NF-κB to stabilize inflammatory signals, resulting in the secretion of pro-inflammatory factors, particularly IL-6, which is associated with AF [68]. Building on this work, other studies demonstrated that the abnormal atrial remodeling can be inhibited by reducing the expression of NF-κB/TNF-α/TGF-β in cardiomyocytes through various methods [69, 70, 71]. In addition, oxidative stress also plays a significant role in the pathophysiology of AF [72], and ROS is a major activator of the NF-κB signaling pathway [73]. The NF-κB transcription factor is sensitive to redox reactions [74]. During oxidative stress, it suppresses transcription of cardiac ${\mathrm{Na}}^{+}$ channels and participates in transcriptional regulation of other ion channels [74]. In cardiomyocytes, an increase in intracellular calcium concentration was observed, attributable to CRP markedly upregulating the expression of sodium-calcium exchanger 1 via the NF-κB signaling pathway [75]. This upregulation further exacerbates cellular oxidative damage [75]. At the same time, myeloperoxidase levels were found to be elevated in the AF group compared to the control group without AF, and myeloperoxidase was independently associated with AF and could be used as an indicator of long-term prognosis [37]. In view of these findings, the NF-κB pathway, as a node of the inflammatory response, seems to be a novel target for the treatment of AF [68, 76].

Many studies have shown that ferroptosis is related to the NF-κB signaling pathway. The classical inflammatory signaling pathways consisting of LOX, COX-2, and NF-κB play an important role in ferroptosis. As mentioned earlier, cells undergoing ferroptosis release DAMP. In response to various stress signals, including DAMPs, NF-κB dissociates and translocates into the nucleus, where it regulates the transcription of other target genes, such as *TNF-α*, *IL-1*, and *IL-8* [4]. At present, a variety of anti-inflammatory drugs down-regulate inflammatory signals such as NF-κB, by inhibiting COX-2, thereby inhibiting inflammatory responses [77, 78]. In the ferroptotic cell GPX4 deficiency as well as the accumulation of iron, intracytoplasmic ROS, and LPO collectively lead to a redox state imbalance, contributing to intracellular inflammatory responses. It has been suggested that GPX4 can inhibit the TNF-mediated activation of NF-κB [50], and also prevent the direct participation of lipid hydrogen peroxide products in the activation of NF-κB [79]. A study has found that in the pathogenesis of obese-related AF, gut microbiota dysregulation and increased lipopolysaccharide (LPS) could affect atrial pathologic remodeling through the activation of ferroptosis and the NF-κB/NLRP3 inflammasome signaling pathway [60]. Thus, inflammation plays a key role in the link between ferroptosis and AF.

#### 3.2.2 TNF-α Activation

The TNF-α peptide is an endogenous inflammatory mediator involved in various cellular processes, including the inflammatory response, cell proliferation, and cell death [80]. Activation of TNF-α is mediated by two surface receptors, TNF receptor type 1 (TNFR1) and TNF receptor type 2 (TNFR2), both of which are expressed in cardiac fibroblasts, cardiomyocytes, and endothelial cells [80]. Pathogen-associated molecular patterns (PAMPs) can simultaneously activate TNFR1 and TNFR2, and subsequently stimulate transcription factors such as NF-κB and activating protein 1 (AP-1). Then, the two major effectors, NF-κB and AP-1 activate TNF-α, resulting in increased inflammation.

At present, it has been concluded that TNF-α can induce AF through structural, electrical, systolic, and autonomic nerve remodeling [80]. Atrial structural remodeling involves cell death (including ferroptosis, necrosis, apoptosis), cardiomyocyte hypertrophy, cardiac fibroblast proliferation, and excessive increase of extracellular matrix, leading to atrial fibrosis [81]. For example, Angiotensin II (Ang-II) is known to cause profibrotic effects by acting on cardiac fibroblasts. Increased PE associated with AF could aggravate Ang-II -induced atrial ferroptosis [45]. It has also been suggested that platelets promote the induction of AF by Ang-II through the release of TNF-α and TGF-β, which interact with cardiac fibroblasts [82]. Specific mechanisms may include increased extracellular matrix proteins, and induction of cardiac fibroblasts to secrete and express bioactive molecules such as TNF-α and TGF-β. In addition, Ang-II can also promote atrial fibrosis by stimulating the synthesis of fibronectin and collagen [82]. The initiation and maintenance of AF includes ectopic triggering activity as well as substrate reentry mechanisms [4]. Fibrosis of the atrial tissue not only leads to slow conduction of electrical signals and the creation of substrates susceptible to unidirectional conduction block, but also promotes the production of new triggers that are likely to initiate the reentry mechanism when these new triggers encounter vulnerable substrates [4, 80]. Ectopic firing is then accompanied by a reentry mechanism, with these factors working together to induce and maintain AF. The mechanism of reentry is also related to ion channel remodeling in atrial cells. It is well known that the pulmonary vein is a common site of abnormal pacing signals in AF, and pulmonary vein isolation is also one of the cornerstones of the treatment of AF [83, 84, 85]. Compared with control pulmonary vein cardiomyocytes (PVCs), TNF-α-treated PVCs had significant delayed afterdepolarization amplitude, smaller sarcoplasmic reticulum calcium content, and greater diastolic intracellular calcium [80]. These findings suggested that TNF-α impairs the ${\mathrm{Ca}}^{2+}$ metabolism of PVCs and increases the susceptibility to AF from the pulmonary veins, thereby leading to inflammation-related AF [80]. Furthermore, TNF-α may also promote AF by acting on gap links such as connexin 40,43 (CX40, CX43) [86, 87]. One review concluded that TNF-α, as one of the hallmark inflammatory pathways of inflammation and oxidative stress in ferroptosis, may interfere with CX40 and CX43 in the atrial gap [88]. In addition, pinocembrin has also been found to regulate the expression of CX40 and other ion channels in cardiomyocytes by inhibiting inflammation [89].

In the ferroptotic cell, when intracellular GPX4 decreases, or ROS and LPO increases, oxidative stress will activate transcription factors such as NF-κB and pro-inflammatory cytokines such as TNF-α, resulting in the differential expression of various inflammatory factors and chemokines, and ultimately, the occurrence of inflammation. It has been demonstrated that TNF-α can rapidly induce spontaneous release of ${\mathrm{Ca}}^{2+}$ and promote atrial arrhythmias, such as AF, by increasing ROS [90]. Elabela, a novel endogenous apelin receptor ligand expressed in endothelial cells of cardiac micro-vessels, has been found to alleviate ferroptosis, cardiac remodeling and fibrosis by regulating IL-6/STAT3/GPX4 signaling [91]. It was suggested that inhibiting the inflammatory response by alleviating oxidative stress may be a promising strategy for the treatment of inflammation-related AF. Thus, TNF-α, as a core inflammatory factor, plays a crucial yet nuanced role in the interplay between atrial cell ferroptosis and AF.

#### 3.2.3 NLRP3 Inflammasome Activation

In the innate immune system, the NLRP3 inflammasome is the most widely studied member of the NOD-like receptor family. The NLRP3 inflammasome is also one of the most important components of innate immunity and plays a key role in defending the body from pathogen invasion and the pathogenesis of various inflammatory diseases [92]. Activation of the canonical NLRP3 inflammasome pathway consists of two steps: priming and activation [93]. In the priming phase, which partially coincides with the previous NF-κB pathway, cytokine receptors (such as receptors for IL-1 and TNF-α), Toll-like receptors (TLRs), and ligands of NOD-like receptors (NLRs) induce pro-IL-1β and NLRP3 expression via the Myd88- NF-κB pathway described above [76, 78, 79]. When the content of antioxidant substances such as GPX4 decrease, or other factors cause an imbalance of the intracellular redox state, accumulation of intracellular iron, aggravated LPO, and mitochondrial dysfunction will induce the activation of the NF-κB pathway and the assembly of the NLRP3 inflammasome [49, 51, 59, 65]. Once the NLRP3 inflammasome is activated and assembled, it induces pro-caspase-1 self-cleavage and activation, promoting the maturation and pro-inflammatory cytokines such as IL-1β and IL-18 [94]. The active caspase-1 cleaves pro-IL-1β and pro-IL-18, which are inactive precursors of IL-1β and IL-18 [94]. Subsequently, activated caspase-1 also cleaves gasdermin D and exposes its N-terminal domain [94]. This N-terminal domain translocates to the cell membrane to form pores that mediate the release of cellular contents as well as the inflammatory factors IL-1β and IL-18, ultimately leading to cell death [94].

There is now clear evidence that the NLRP3 inflammasome plays a causal role in the pathogenesis of AF [5, 93]. The NLRP3 inflammasome is also associated with ferroptosis. GPX4 is an irreplaceable inhibitor of ferroptosis, and it is also known to inhibit activation of the NLRP3 inflammasome [94]. A study has found that a high-fat diet may induce AF through an intestinal flora imbalance leading to lipopolysaccharide production, which could result in ferroptosis of atrial cells and the enhancement of the TLR4/NF-κB/NLRP3 inflammasome signaling pathway [60].

A previous study established the pathophysiological mechanism of the NLRP3 inflammasome in atrial cells in the pathogenesis of AF [95]. It was found that the ryanodine receptor type 2 (Ryr2) ${\mathrm{Ca}}^{2+}$ release channel, mediated by sarcoplasmic reticulum dysfunction, played a key a role in delayed afterdepolarization (DAD) triggered activity [96]. During diastole, the Ryr2 ${\mathrm{Ca}}^{2+}$ release channels are implicated in abnormal ${\mathrm{Ca}}^{2+}$ leakage, potentially contributing to atrial ectopic activity and reentry substrate mechanisms [4, 96, 97]. In atrial cells, aberrant NLRP3 signaling led to increased expression of (Ryr2) ${\mathrm{Ca}}^{2+}$ release channel, increasing ${\mathrm{Ca}}^{2+}$ release, and precipitating ectopic firing through DAD [95]. Moreover, the NLRP3 inflammasome in atrial cells mediates calcium-dependent protein kinase II activation [93, 97]. This activation promotes sarcoplasmic reticulum calcium release and exacerbates mitochondrial dysfunction, accompanied by a build-up of ROS [93, 97]. Such changes result in DADs and triggered activity, perpetuating a cycle of inflammatory signaling and cellular dysfunction [93, 97]. Hyperactivated NLRP3 signaling enhances transcription of *potassium voltage-gated channel subfamily A member 5* (*Kcna5*), resulting in enhanced *$I_{k}$^+^* current and the formation of reentry substrates [95]. Additionally, overactive NLRP3 signaling promotes caspase-1 cleavage, which activates cardiac fibroblasts and stimulates the recruitment and secretion of inflammatory cytokines. This cascade of events, along with the background inflammatory factors and excessive secretion by atrial fibroblasts facilitate the development of a substrate that maintains AF [95].

Experimental studies have shown that the activation of the NLRP3 inflammasome, when restricted to cardiac fibroblasts, can lead to atrial inflammatory changes, atrial fibrosis, and AF [98]. Specifically, cardiac fibroblast-restricted NLRP3 inflammasome activation can intensify the activity of cardiac fibroblasts, over-expression of connexin, atrial tissue fibrosis, and impair autonomous cell function [98]. Pinocembrin has been reported to alleviate atrial fibrosis and electrical remodeling by reducing the expression of NLRP3, caspase1, IL-1β and other inflammatory factors [89]. Furthermore, the link between gut microbiota and the atrial NLRP3 inflammasome may be a reasonable target for the treatment of age-related AF [99]. In conclusion, the NLRP3 inflammatory signaling pathway is one of the key factors in the pathogenesis of inflammatory AF. The NLRP3 inflammasome has been extensively studied in the pathogenesis of AF. However, as one of the core biological factors of the inflammatory response, the specific role of the NLRP3 inflammasome and its related inflammatory pathways in the relationship between ferroptosis and AF needs to be further studied.

### 3.3 Role of ROS in Mitochondria between Ferroptosis and AF

Mitochondrial dysfunction is a significant feature of ferroptosis, contributing to the occurrence and progression of AF. A recent review concluded that the effect of the NLRP3 inflammasome on ferroptosis was achieved by changing the level of ROS [100]. Similarly, the induction of the NLRP3 inflammasome by abnormal intracellular ROS levels also contributes to ferroptosis [100]. The excessive production of ROS is not only related to the inflammatory response, but is also associated with the degree of mitophagy in ferroptotic cells. Mammalian target of rapamycin (mTOR), a serine-threonine kinase, is an important regulator of cell metabolism and growth, and also participates in the regulatory mechanism of ferroptosis. Tristetraprolin and mTOR are participants in intracellular iron homeostasis, regulating iron-containing genes and the mRNA stability of the transferrin receptor 1 [101].

An experimental study has found that the ablation of the NLRP3 inflammasome can improve a series of aging-related metabolic features (including cardiac aging-related inflammation and fibrosis) [102]. This may be related to the activation of autophagy, reduction of the insulin-like growth factor 1 (IGF-1) pathway and the phosphatidyl inositol 3 kinase/protein kinase B/mammalian target of rapamycin (PIK3/AKT/mTOR) pathway [102]. Another study found that mTOR was also a profibrotic signal involved in the excessive inflammatory response and fibrotic remodeling of AF through the C-X-C chemokine ligand 12/C-X-C chemokine receptor type 4 (CXCL12/CXCR4) axis [103]. Further research also links mTOR activation with fibroblast proliferation, increased fibroblast-to-myofibroblast transformation, and cardiac collagen synthesis [104], highlighting mTOR’s involvement in AF. Over-activated mTOR stimulates the NLRP3 inflammasome, recruits pro-inflammatory factors, and contributes to AF pathogenesis by promoting fibrosis and altering atrial cell electrophysiology. Consequently, mTOR has emerged as a potential therapeutic target for AF, with its inhibition potentially reducing AF incidence.

In contrast to these favorable results, a study found that when mTOR was inhibited in cardiomyocytes, iron accumulation occurred in the cells, leading to iron overload [100]. This inhibition of mTOR promoted mitophagy, which often coincides with cell autophagy, triggering the degradation of ferritin and potentially inducing ferroptosis [100]. Sirtuin3, a classical ${\mathrm{NDA}}^{+}$-dependent mitochondrial protein deacetylase, is responsible for deacetylating mitochondrial proteins subjected to oxidative stress and regulating cellular metabolism [105]. Research has shown that Sirtuin3 enhanced the phosphorylation of AMP-activated protein kinase (AMPK), which inhibited the activity of mTOR and ultimately promoting both autophagy and ferroptosis [105]. Researchers also found that the mTOR pathway was inhibited in AF patients aged 60 to 70 years, with mTOR-related genes downregulated in atrial tissue [106]. These results suggest that inhibition of mTOR and its related pathways may contribute to cardiac ferroptosis and the onset of AF. Therefore, mTOR may be a pivotal link between ferroptosis, inflammation and AF.

The experimental evidence regarding the roles of mTOR and autophagy in the context of ferroptosis, inflammation, and AF is not straightforward. To address these contradictory findings, it is hypothesized that the level of mTOR inhibition and the degree of mitophagy act as a double-edged sword. Under high stress conditions, such as decreased GSH intake, decreased GPX4, and iron overload, cells experience a significant imbalance in intracellular redox metabolism, leading to intracellular LPO. At the same time, large amounts of ROS are also produced within mitochondria. Mild autophagy can be beneficial for cells, helping to remove necrotic organelles and substances exerting anti-inflammatory effects. Mitophagy, the targeted removal of damaged mitochondria, can decrease ROS production and subsequently inhibit the activation of the NLRP3 inflammasome [102].

mTOR, a critical regulator of inflammation and autophagy, exhibits contrasting effects depending on its activity level. Overactivation of mTOR induces the recruitment of inflammatory factors and promotes fibrosis [103, 104]. Conversely, mild inhibition of mTOR induces mitophagy, which aids in ROS clearance and provides cellular protection [102]. However, excessive inhibition of mTOR will lead to severe mitophagy, which will lead to cell death, ferritin degradation and ferroptosis [105]. Additionally, studies have also found that GPX4 overexpression can improve mitochondrial dysfunction [107]. There was evidence that inhibition of GPX4 resulted in LPO as well as ROS accumulation and promoted autophagy [107]. Thrombospondin 1 is thought to inhibit autophagy [107]. Overexpression of GPX4 promoted the production of thrombospondin-1 [107]. Therefore, GPX4 could block autophagy by releasing thrombospondin 1, which eventually attenuated cardiac fibrosis [107]. GPX4 was also thought to alleviate fibrosis by reducing intracellular LPO and inhibiting the TGF-β pathway [107]. In summary, GPX4 protects against ferroptosis while also alleviating fibrosis. The relationship between inflammation, ferroptosis and AF is multi-dimensional, and there are many key nodes that dominate the role of ferroptosis in the pathogenesis of AF.

### 3.4 Discussion

In our study of the connection between inflammation and ferroptosis in AF, we focused on the activation of the NF-κB pathway, TNF-α and the NLRP3 inflammasome as representatives of inflammatory response to elucidate the relationship between ferroptosis and AF. Atrial inflammation is one of the important factors leading to the occurrence and maintenance of AF. Although AF is closely related to atrial fibrosis induced by atrial inflammation, there are two different types of atrial fibrosis: “reactive” fibrosis and “reparative” fibrosis [108]. Moreover, these two types of fibrosis have different effects on the induction of AF. “Reactive” fibrosis is characterized by thickening of the normal fibrous connective tissue surrounding the muscle bundles while the muscle bundles itself is structurally intact [108, 109]. When myocardial cell necrosis occurs, such as in a myocardial infarction, apoptosis, and ferroptosis, the replacement of dead cardiomyocytes by fibrous tissue is the important pathological feature of “reparative” fibrosis which is largely irreversible [108, 109, 110].

In contrast, “reactive” fibrosis can impair cardiac longitudinal conduction, but only when thicker interstitial collagen chains are present between atrial myocytes [111]. “Reparative” fibrosis, however, creates conduction blocks due to cell death and disrupted muscle bundles. Furthermore, the uneven distribution of cardiomyocytes and collagen fibers disrupts the continuity of atrial electrical signals, potentially leading to local circuit formation and promoting reentry mechanisms [111, 112]. In addition to irreversible atrial fibrosis, abnormal atrial ion channels caused by inflammation are another important mechanism leading to the occurrence of AF. It can be concluded that ferroptosis, as a type of cell death related to the heart, plays a detrimental role in inducing the inflammatory response and atrial fibrosis.

Given the central role of iron metabolism in cardiac health, we sought to identify the key molecules in the inflammatory signaling pathway associated with ferroptosis, to explore new targets for AF treatment, and to search for possible highly affinity and selective inhibitors for these targets. A study indicated that ferroptosis caused by iron overload is involved in new-onset AF in sepsis, a systemic inflammatory response, with ferroportin potentially playing a key mediating role [61]. *In vitro* experiments on atrial cells exposed to excessive alcohol revealed an increased susceptibility to AF, marked by elevated levels of ferroptosis-promoting molecules such as P53 and ACSL4 [62]. Icariin was shown to inhibit ferroptosis in atrial cells, reducing atrial inflammation and oxidative stress by activating the atrial SIRT1-Nrf-2-HO-1 signaling pathway [62].

Contrasting our hypothesis that ferroptosis leads to AF onset, another study proposed that AF could induce ferroptosis, especially in maintaining persistent AF [63]. This research involved rapid atrial pacing in experimental dogs, leading to the secretion of exosomes by atrial cells and cardiac fibroblasts [63]. The exosome inhibitor GW4869 reduced atrial inflammation and collagen deposition in the experimental group. In addition, they found that exosomes secreted by cardiac fibroblasts promoted ferroptosis in h9c2 cells, and that miR-23a-3p encapsulated in the exosomes may be the key molecule responsible for ferroptosis [63]. Therefore, specific inhibitors of miR-23a-3p can be regarded as a potential therapeutic target to disrupt the ferroptosis-AF cycle [63]. The above evidence strongly suggests that ferroptosis and AF are closely linked and may be complemented by atrial inflammation. These findings offer new potential targets, such as inhibiting signaling molecules or inflammatory factors in ferroptosis-related pathways. In conclusion, ferroptosis is intimately linked with AF, atrial inflammation, subsequent atrial fibrosis, and ion channel abnormalities, all of which play significant roles in this intricate process.

## 4. Conclusions

In this review, we discussed ferroptosis, inflammation and AF in detail, striving to elucidate the relationship between inflammation and ferroptosis. As one of the results of ferroptosis, inflammation may be involved in the pathogenesis of AF. We also reported the role of mitochondria and autophagy in the ferroptosis-AF nexus, highlighting several key enzymes and inflammatory signaling pathways. This approach offers a more comprehensive theoretical framework for understanding the association between ferroptosis and AF, especially from the perspective of ferroptosis. In addition, the detection of myocardial iron metabolism and inflammatory factors can be used as targets for the treatment of AF. At present, ferroptosis is recognized as a novel mode of cell death and has attracted considerable attention. Research in this area has predominantly focused on conditions such as cardiomyopathy, myocardial infarction, and ischemia-reperfusion. However, the exploration of ferroptosis’s connection with AF remains relatively uncharted territory. At present, it has been shown that AF is associated with ferroptosis, including the bioactive molecules related to ferroptosis [36]. Therefore, a multi-dimensional analysis of ferroptosis in the context of AF is imperative. Looking ahead, we anticipate that future experimental research will further investigate the role of inflammation in the interplay between ferroptosis and AF, and actively pursue new therapeutic targets for AF treatment.
